# Conceptual framework and rationale

**DOI:** 10.1186/1475-2875-8-S2-S1

**Published:** 2009-11-16

**Authors:** Alan S Robinson, Bart GJ Knols, Gabriella Voigt, Jorge Hendrichs

**Affiliations:** 1 Entomology Unit, FAO/IAEA Agriculture and Biotechnology Laboratory, IAEA Laboratories, A-2444 Seibersdorf, Austria; 2 Div. Infectious Diseases, Tropical Medicine & AIDS, Academic Medical Center, F4-217, Meibergdreef 9, 1105 AZ Amsterdam, The Netherlands and K&S Consulting, Kalkestraat 20, 6669 CP Dodewaard, The Netherlands; 3 IAEA Laboratories, A-2444 Seibersdorf, Austria; 4 Joint FAO/IAEA Programme of Nuclear Techniques in Food and Agriculture, IAEA, Wagrammerstrasse 5, A-1400 Vienna, Austria

## Abstract

The sterile insect technique (SIT) has been shown to be an effective and sustainable genetic approach to control populations of selected major pest insects, when part of area-wide integrated pest management (AW-IPM) programmes. The technique introduces genetic sterility in females of the target population in the field following their mating with released sterile males. This process results in population reduction or elimination via embryo lethality caused by dominant lethal mutations induced in sperm of the released males. In the past, several field trials have been carried out for mosquitoes with varying degrees of success. New technology and experience gained with other species of insect pests has encouraged a reassessment of the use of the sterility principle as part of integrated control of malaria vectors. Significant technical and logistic hurdles will need to be overcome to develop the technology and make it effective to suppress selected vector populations, and its application will probably be limited to specific ecological situations. Using sterile males to control mosquito vector populations can only be effective as part of an AW-IPM programme. The area-wide concept entails the targeting of the total mosquito population within a defined area. It requires, therefore, a thorough understanding of the target pest population biology especially as regards mating behaviour, population dynamics, dispersal and level of reproductive isolation. The key challenges for success are: 1) devising methods to monitor vector populations and measuring competitiveness of sterile males in the field, 2) designing mass rearing, sterilization and release strategies that maintain competitiveness of the sterile male mosquitoes, 3) developing methods to separate sexes in order to release only male mosquitoes and 4) adapting suppression measures and release rates to take into account the high reproductive rate of mosquitoes. Finally, success in area-wide implementation in the field can only be achieved if close attention is paid to political, socio-economic and environmental sensitivities and an efficient management organization is established taking into account the interests of all potential stakeholders of an AW-IPM programme.

## Background

Most insect control methods, both past and present, rely on the modification of some component of the insect's environment; in the majority of cases this means the use of insecticides. The realization that compromising the integrity of the hereditary machinery, through manipulation of the reproductive cells to induce genetic sterility, could also provide a method of pest control was a considerable conceptual leap [1]. Targeting the genome of pest insects to mitigate against their deleterious effects is currently undergoing a renaissance, especially in mosquitoes, with much attention being focused on using transgenesis to manipulate vector competence [2]. This approach, however, still has to overcome major regulatory hurdles and will require the identification of efficient effector genes and drive mechanisms to ensure the spread of any refractory gene. However, transgenic approaches may also contribute to the development of male-only strains [3] and provide alternative ways to sterilize mosquitoes [4].

When the concept of using sterility for insect control was first considered, entomologists were not aware of any treatment that could be given to insects, which following their release and mating with wild insects, would lead to the induction of sterility in individuals of the field population. Muller's discovery that ionizing radiation could induce dominant lethal mutations [5] was only appreciated by entomologists in the 1950's [6] and this led to the now well known and very impressive elimination of the New World screwworm *Cochliomyia hominivorax*, from the Southern States of the USA, Mexico and all of Central America and Panama [7], using what has come to be called the Sterile Insect Technique (SIT).

This initial success for New World screwworm was quickly followed by many attempts to develop similar approaches for mosquitoes, with varying degrees of success [8]. Nevertheless, the release of sterile insects remains the only genetic technique that has been successfully implemented for the control of major insect pests over large areas [9].

### What is a sterile male?

The induction of genetic sterility in the females in the field population remains the key requirement for success of the SIT. Sterility is caused by dominant lethal mutations in the sperm of the released males resulting from radiation. A dominant lethal mutation is one that leads to the death of the developing zygote, in this case the embryo, irrespective of the genetic contribution of the other gamete. A sterile male has therefore to mate and transfer viable sperm and also accessory fluid of the appropriate quality and quantity to ensure appropriate female behaviour. This means that the definition of a sterile male, in terms of anopheline SIT, is very narrow and it does not include males that are aspermic, or which do not transfer viable sperm, or which do not elicit the correct behavioural response in the female, or which in any other way fail to convince the female that she has been mated by a "normal" male. In fact, the sterile male could be considered to be not sterile himself; the sterility must only be effective in the following generation when his sperm is used by the wild female to fertilize her eggs. The sterile male is simply a carrier of genetically compromised sperm from the rearing facility to the wild females in the field.

### Effectiveness of SIT at low insect densities and integrated approaches

The first modellers to propose operational strategies for the use of sterile insects quickly realized that natural populations of pest insects can be present in such high numbers and often have such a high rate of population increase that it is very difficult to conceive the rearing of such high numbers of insects [10]. The SIT is clearly not a stand-alone technology and has to be integrated with other methods that are much more effective at suppressing pest populations that are at very high densities. However, sterile insects can be very effective at low pest densities where the technique exploits the ability of insects to find mates in the field. Seasonality in pest population densities offers opportunities to maximize the ratio of released sterile to wild insects. Suppression techniques can be integrated in time as well as space. For example, sterile male mosquitoes can be released at the same time that insecticide treated bed nets or indoor residual spraying are being used as different components of the female life cycle are being targeted i.e. mating, feeding and resting, respectively.

### Area-wide integrated pest management

To be most effective, released sterile insects have to target a discrete vector population on an area-wide basis, but strict isolation is obviously not a requirement considering the success of the New World screwworm programme. The area-wide integrated pest management (AW-IPM) approach is not a concept that is restricted to the use of sterile insects [11] and has been utilized in successful vector control programmes e.g. *Simulium damnosum *in West Africa [12] and *Anopheles gambiae *in Egypt [13]. The AW-IPM approach that includes SIT entails releasing sterile insects over the whole area where the vector population is present and not just where the vector is responsible for malaria transmission. The need for this AW-IPM approach is both the strength and, to some extent, the weakness of the technology. The strength is that pest suppression can be more efficiently achieved and under certain ecological conditions and programme goals can lead to the sustainable elimination of a local population of a particular pest species. The weakness is that it requires techniques to be available that can effectively suppress populations on an area-wide basis i.e. in areas where there is no human population or where there is very difficult terrain. These requirements tend to give the impression that the area-wide approach is a "top-down" technology that is difficult to reconcile with the prevailing philosophy of community-based strategies for vector control. In reality however, not only is community acceptance required, but also extensive coordination among all stakeholders within the target area. The AW-IPM approach requires therefore attention to political, economic and environmental realities and sensitivities, and excellent communication with the public and local authorities. It is also management intensive and requires an almost military-like structure for programme implementation. Until recently, the AW-IPM approach, opening up the possibility of efficient suppression or in some cases elimination of local populations of insect disease vectors, has been a controversial subject and will continue to be so as it seems to reflect deep philosophical differences in how a "disease" and its "control" are perceived by the various groups and other stakeholders not directly exposed to the disease [14]. However, recently the concept of global eradication of malaria is under discussion [15].

## Key issues for success

### Mosquito population dynamics and regulation

Perhaps the most contentious and difficult issue to deal with in terms of integrating the release of sterile insects into mosquito control relates to the high reproductive potential of mosquitoes and level of density dependent population regulation in the field; parameters over which very limited control can be exercised. In order to cause a decline in population size, sufficient sterility has to be introduced into the population to reduce the rate of population increase, R_0_, below one. When R_0 _stays below one for several generations then the population will eventually disappear. Continued release of sterile males in the same numbers will increase the rate at which R_0 _is reduced and population decline will accelerate. The correct estimate of R_0 _in the target population, as well as its size following pre-release suppression is required, in order to identify the level of sterility that is required to initiate a population reduction. This information can then be translated into the number of sterile males that need to be released if their competitiveness and level of induced sterility is known. In mosquitoes, values of R_0 _vary greatly but can be as high as ten indicating that over 90% sterility will need to be induced in the target population to cause a population decline. If the released males were fully competitive and fully sterile then at least ten times more sterile males would need to be released than wild males present in the field. Since full competitiveness will never be achieved, this results in the need to have even larger over-flooding ratios in order to cause a population decline.

Density dependent regulation will also impact on the effect that induced egg sterility will have on the final population size in the field. Density dependent regulation of larval mortality can strongly counteract any reduced egg hatch as a result of the release of sterile males. If 50% of larvae die due to density related effects then reducing egg hatch by 50% will have no impact on the final size of the adult population.

It is essential that baseline data on all these population processes in the field are collected on a temporal and spatial basis during a thorough feasibility study to assess the potential for an AW-IPM programme which includes SIT. These data are then used to determine the degree of the required pre-release population suppression and the number of sterile males required for release; this information is also used as the basis for the design of a mass rearing facility of the appropriate size. It will also be essential to ascertain whether previously fertilized females can enter the target area, as these females are largely immune to sterile releases. The extent of such immigration will determine the need for, and width of, buffer or barrier zones and degree of suppression of the vector population in the target area that can be expected [16]. In addition to using sterility for population reduction, perturbing the fertility of a natural population through the induction of sterility is a very powerful tool to study the underlying dynamics of vector populations [17].

### Field competitiveness of mass-reared mosquitoes

The requirements that a sterile male must meet in order to be effective are considerable. The sum of all these requirements is generally called "competitiveness" and protocols must be developed so that accurate values of field competitiveness of sterile male mosquitoes can be calculated. Without this, release strategies cannot be developed, assessment of efficacy becomes impossible and there is a great danger of failure, as large numbers of sterile insects could be released without any impact on the field population. Deficiencies in the competitiveness of sterile males are probably the single most significant technical reason for failure. Laboratory-based protocols to measure competitiveness are at best inadequate and at worst misleading. The only relevant measure of the competitiveness of sterile male mosquitoes can be made in the field when in competition with males from a field population. However, this is operationally difficult to do but can be approached by using a semi-field cage system [18,19] and it should be validated during a field programme. This type of evaluation should be made routinely and especially following any change in the mass rearing, transport and release procedures as it will enable those processes or protocols that have the most impact on competitiveness to be identified and perhaps modified.

### Retaining field competitiveness in mass reared mosquitoes

How can competitiveness be best maintained in mosquitoes that are mass reared for many generations under highly artificial conditions, subjected to intrusive handling and sterilization procedures, marked with a fluorescent dye, transported from a rearing facility to the release site and eventually released, maybe from an aircraft? All these processes will inevitably impact negatively, and to different degrees, on field competitiveness. However, probably the major factor affecting field performance of sterile males is the accumulation of laboratory-adapted genotypes in the mass reared colony. In the colony the biotic and abiotic environments are always totally different from those in the field so that there is rapid, severe and continuous selection for genotypes that enable the colony to be most productive under laboratory conditions. Paradoxically, these may be the very genotypes that have the most serious negative consequences for the competitiveness of the insect in the field. Is it possible to reconcile these two opposing forces to produce a sterile mosquito that fulfils most of the requirements for SIT? The answer may be yes, with some reservations, based on new mass rearing strategies being developed in other insects e.g. the Mediterranean fruit fly *Ceratitis capitata *[20,21].

Mass rearing of insects is usually carried out by maintaining a very large cycling population, from which a proportion of the insects can be harvested for sterilization and release. This large cycling population is initiated at every generation from insects that have been through the mass rearing process, which leads to the accumulation of the laboratory-adapted genotypes over time. This can be prevented by changing the mass rearing strategy from cyclical to directional. In directional mass rearing, a relatively small colony, the stock or mother colony, is maintained under as natural conditions as possible and from this colony eggs are regularly collected to begin the mass rearing process which can take up to 4-5 generations before sufficient insects are available for sterilization and release. Very importantly, no insects that have been through the intensive mass rearing process are returned to the mother colony as all are sterilized and released. The size of the mother colony and the number of generations that are required to produce sufficient insects for release depends on the programme requirements and the insect's fecundity. Using directional mass rearing, the mother colony can be maintained under any set of desired conditions, even in a secure greenhouse, providing eggs can be easily collected. It is also possible to replace a strain without major impact on production. Obviously, this type of rearing results in a trade off between efficiency of mass production and the competitiveness of the insect produced for release; the more natural the conditions for the mother colony the less productive the insects will be when subjected to the small number of generations of mass rearing necessary to produce sufficient insects for release. Considering the essential role that competitiveness plays in success or failure of a sterile release, much more attention will need to be focussed on quality rather than quantity in developing the SIT for mosquitoes. Effective mass rearing and colonization of mosquitoes is further discussed in [22].

### Effective sterilization procedures

The use of chemosterilants in the successful El Salvador pilot programme [23] is unlikely to be repeated due to concerns about environmental contamination, and currently radiation is the method of choice. Radiation induces a large suite of different dominant lethal mutations in irradiated sperm, and the higher the dose the more likely it is that every sperm carries at least one dominant lethal mutation. This means that a dose needs to be identified that causes at least one dominant lethal mutation in every sperm and consequently leads to full sterility in the treated male. However, as the chance that every sperm carries a lethal has a certain probability and by sampling an infinite number of eggs fertilized by irradiated sperm, there is always the probability, however small, that one egg may be fertile and hatch. This makes it very difficult to talk about a sterilizing dose in absolute terms and raises the question as to what is the appropriate dose to use in an operational programme. As described above, the amount of induced genetic sterility in field females is the key determinant of success or failure, and this amount is determined not just by the level of induced sterility in the male but also by its competitiveness in the field. The sterility induced in the field females is the product of the induced sterility in the male multiplied by its competitiveness in the field. Achieving full sterility through the use of a high dose of radiation, at the expense of greatly reduced competitiveness, is generally not the best strategy. A recent publication describes this trade-off in more detail and provides guidelines as to how best to decide on the dose of radiation that is given to males for release in an SIT programme [24].

Most control programmes with an SIT component irradiate pupae as that particular stage is convenient for handling, however often this results in reduced competitiveness of the males compared to when adults are irradiated [25]. This is because the later during development the radiation is given, the less somatic damage is induced. For mosquitoes however, pupal irradiation would appear to be difficult considering the aquatic environment and the relatively short pupal period. Irradiation of adult male mosquitoes will require protocols to collect and anaesthetize large numbers of male insects for confinement during the short radiation process. The use of nitrogen may be a solution to this as it can be used for anaesthetising mosquitoes and has the added benefit that it gives some protection against somatic damage induced by the radiation [26]. More details on radiation biology of mosquitoes can be found in [27].

Molecular approaches to sterilizing insects have been proposed [28-31] and these systems are discussed more fully in [4]. These approaches require conditionality of the expression of certain genes so that a fertile colony can be reared in the laboratory and that sterility is only expressed in the eggs of wild females when mated with the released transgenic males. These systems rely on transgenesis, of which very little is known in terms of its suitability as an effective and safe technology out of the laboratory environment, so much work and evaluation has still to be done before a workable system can be tested on a meaningful scale. One potential advantage that radiation has is built in redundancy as a large majority of sterile sperm will carry more than one dominant lethal mutation and each mutation is different making it impossible to the field population to develop any sort of resistance (but see below). With molecular approaches sterility may be determined by a single genetic factor that is identical in all males, and a natural variant that compromises the effectiveness of the factor will very rapidly spread through the field population. There is also the issue of stability of the transgene construct during the mass rearing process.

### Producing only males

Sex separation leading to the release of only sterile males is essential in mosquitoes since release of sterile females would contribute to disease transmission. Several techniques have been tested to achieve this [32] with one being used in an operational mosquito programme [33]. Specific approaches using classical and transgenic techniques to achieve this in mosquitoes are detailed in [3]. To provide the degree of accuracy required for sex separation and the large numbers of insects that need to be processed, it will probably be necessary to rely on some form of genetic or molecular approach as opposed to natural sexual dimorphic characters such as pupal size. It is probably also essential that separation takes place at the population level and not at the individual level. In other words, systems which rely on automated recognition and sorting of individual pupae or larvae will probably not be useful for operational programmes. For economy of mass production, sex separation should take place as early as possible during development. Any biological sexing system, whether based on classical or transgenic approaches, will always be subjected to the normal genetic processes that continuously take place in the genome. These processes can act to compromise the sexing efficiency and lead to a rapid destabilization of the strain, the effects of which can be magnified under large scale cyclical mass rearing where selection pressure on any novel genotype will also play a major role in determining the speed at which any particular strain loses its sexing efficiency [34]. The directional mass rearing system described above needs to be a component of a systems approach to maintain genetic integrity of a strain in the mother colony which, being small, can be routinely monitored for the occurrence of unexpected genotypes that can then be removed.

## Solutions to identified problems

### Understanding mating behaviour

Mating competitiveness of released sterile male mosquitoes can only be defined in relation to the normal mating behaviour of wild males in the field. While many programmes have succeeded with little mating knowledge, the effectiveness of programmes increases with understanding of this behavioural component and as protocols are in place to monitor this in mass reared mosquitoes [35]. These techniques may involve analysis of video recordings, aggregation and sex pheromones, visual cues and swarming in time and space. It will also be important to be able to 1) monitor the numbers of male mosquitoes in the field in order to assess the ratio of sterile males to wild males, 2) develop effective traps to monitor the presence of released males and dynamics of the target population, 3) estimate population size, 4) study vector population dynamics and dispersal and 5) assess the level of reproductive isolation among the different populations.

### Measuring sterility in the field

As induced sterility in the field females is the link between the released sterile males and the eventual reduction in population size, a very important parameter is the measurement of sterility in a field population subjected to sterile male releases. By having data on the ratio of sterile to fertile males and the level of sterility in field females, accurate estimates can be made of male mating competitiveness. Combining these data with estimates of population size enables valuable information to be obtained on the reproductive potential of the field population and the overall trend in population size. Estimates of sterility can be measured directly if female mosquitoes can be easily trapped alive, blood fed, egged and hatchability measured. This will not be straightforward for *Anopheles* mosquitoes when they occur at low densities although it was done quite effectively during the SIT programme in El Salvador [32]. An alternative to this would be to label the released males with an agent that is incorporated into the accessory fluid and transferred during mating in an amount that can be monitored in the dissected spermathecae of field females. Stable isotopes may offer this possibility [36]. Recently, using transgenesis, it has been demonstrated that sperm in the spermatheca of females can be visualized using a fluorescent protein [37]. These marking systems could also be used to differentiate released males from wild males (see below).

### Resistance to SIT

Insects have shown themselves to be extremely effective in dealing with environmental constraints aimed at their control; this is especially so for chemical modification of the environment i.e. insecticides. Can insects develop resistance to sterility induced by the release of sterile males? As stated above the radiation-sterilized males carry an almost infinite variety of "sterilizing factors" i.e. dominant lethal mutations, in the irradiated sperm and it is inconceivable that females in the field could become resistant to the lethal effects of these mutations. However, could female mosquitoes become "resistant" to the carrier of the mutations, the sterile male, as a result of selection? For this to happen, natural selection would have to favour female mosquitoes that preferentially mate with fertile field males and this requires that there has to be a) a fitness cost for the female for mating with the sterile male, b) a recognizable trait in the sterile males and c) heritability of the recognition of the trait in the field females. Condition a) is always met as the fitness cost to mating with the sterile male is 100%. Condition b) is more difficult to generalize as it will depend to a large extent on the mating behaviour of a particular species. In species where female choice plays a major role it is likely that there would be an increased chance for resistance to occur than when the male is the major determiner of mating success [38].

In anopheline mosquitoes, where males form swarms and receptive females are attracted to the swarms, it is of course essential that the sterile males participate effectively in the swarm. These swarms are not considered to be leks as there is no male aggression before the female arrives and no courtship after the female mosquito arrives in the vicinity. With this type of mating behaviour, whereby females in close proximity to the swarm are aggressively mated by males leaving the swarm, the development of behavioural resistance would seem to be less likely than in lekking species with a clear female mate choice.

### Improving rearing and release technology

As indicated above, rearing protocols can be developed which strive to maintain field competitiveness in the mass reared sterile mosquitoes. However, the rearing systems themselves will need major modification to ensure stable and predictable production levels can be guaranteed. Large adult holding and oviposition cages will be needed which incorporate *in vitro *blood feeding systems and easy harvesting of eggs. Closed egg incubation and larval rearing systems will be required (perhaps using aquaculture principles) which guarantee synchronous development of the larval instars and from which mature pupae can be easily harvested for subsequent adult emergence, sterilization and release. During this development much attention will need to be paid to larval food requirements to ensure predictability and synchrony of development. Innovative mosquito marking methods [39] using rare earth elements, stable isotopes or proteins can be included in the protocols for sterile male handling. Releasing large numbers of sterile male mosquitoes in an AW-IPM programme is a daunting task as this may require their release from an aircraft. This has so far not been done with mosquitoes but is commonplace in programmes with other more robust insects. Efficient containment of large numbers of sterile male mosquitoes in confined volumes will be required and current shipment and release technology will need to be adapted to deal with the fragility of mosquitoes. However, the successful pilot programme in El Salvador used ground release of sterile pupae and this may indeed be more appropriate in many situations where the target population is delimited and accessible.

### Improving quality control protocols

Quality control (QC) protocols are indispensable to any SIT programme and they are composed of three components: the production (i.e. equipment, materials, the environment etc), the process, (i.e. the physical components involved in production, sterilization, transport and release) and the product, (i.e. the sterile insect) [40]. The most important component of product QC will be the assessment of field or semi-field competitiveness of the released male, however also in the rearing facility, systems for the routine measurement of reproductive parameters must be developed. In comparison to other species where SIT is being used, the inputs in terms of consumables are few and relatively easy to control with a guaranteed supply of quality blood being the major concern. To feed one million female mosquitoes about 10 litres of blood per week would be needed.

### Measuring impact of sterile releases

Entomological monitoring as described above should be able to describe the impact of the sterile male release on mosquito population density in the field. Much more difficult will be to assess the impact on the disease picture. If a programme involving sterile male releases is relatively successful, entomological parameters such as the sporozoite rate or the entomological inoculation rate would be very difficult to measure. In fact, the vector densities would be so low that adequate sample sizes for infection rates, anthropophilic index and daily survival rate estimates would be very difficult to obtain. Under such conditions, a better way to assess the impact would be to determine in addition to vector densities, the morbidity and mortality parameters over an adequate period of time, although this will also not be straightforward.

## Feasibility studies for *An. arabiensis *SIT

The Joint FAO/IAEA Programme of Nuclear Techniques in Food and Agriculture has for many years been involved with the development and use of sterile insects to control several key agricultural pests such as several species of fruit flies and tsetse flies (Glossinidae). Success in these programmes has resulted in many requests to assess the feasibility of the technique for malaria vectors. As a response to this, a mosquito R & D programme based at the FAO/IAEA Agriculture and Biotechnology Laboratory, Seibersdorf, Austria has been initiated to assess the field feasibility of integrating the SIT into *Anopheles arabiensis *control. The project has long-term objectives and seeks to establish basic capabilities in selected locations in sub-Saharan Africa to develop appropriate SIT-based methodologies and to assess their feasibility and applicability for mosquito control. The enabling technologies that need to be developed and implemented have been outlined and discussed above. The initial activities in the field have focused on the identification of potential field sites where the SIT technology can be evaluated. This is further discussed in [41].

In Sudan, two field sites along the great bend of the Nile have been identified, Merowe and Dongola, and a GIS-based mosquito data collection system has been introduced. Two automatic weather stations have been installed at each site and satellite data for the two sites have been obtained. GPS based data loggers have been programmed and a two year larval survey has been carried out [42]. A population genetic survey of the *An. arabiensis *populations in the two project areas has also been completed. In 2007, the first releases of sterilized male mosquitoes produced from genetic sexing strain were made and plans are being developed to construct a mosquito rearing facility in Khartoum capable of producing one million sterile males/day.

In this part of Sudan, malaria affects the livelihoods of over 0.5 million people, and is responsible for 30% of all hospital admissions. All stakeholders in the region are aware of, or actively contributing to, the project. The project receives full backing from the Ministry of Science and Technology and Ministry of Health alongside the National Malaria Control Programme and Tropical Medicine Research Institute in Khartoum. Recently a donors meeting succeeded in attracting one million USD for the project [43].

## Conclusion

Solving all the technical and scientific constraints described above is a major challenge for any SIT programme. However in itself, this is not sufficient to ensure success. AW-IPM programmes of any type are extremely management intensive and do require long-term commitment and a very inclusive and integrated structure to ensure that all the different stakeholders are fully involved at all stages of the programme. Lessons learned during the WHO/ICMR programme in India are a dire warning as to how sound scientific programmes can be blown completely off track by inadvertent or, as in that particular case, deliberate misuse of information [44].

## Competing interests

The authors declare that they have no competing interests.

## Authors' contributions

ASR wrote the paper and BGJK, GV, and JH made subsequent contributions and edited the chapter.

**Table 1:** Suggested further reading

***Historical perspective***	
First SIT programme in Florida against the screwworm	[45]
Biography of E.F. Knipling with references to his most important papers	[46]
***Models***	
Use of sterile insects with reduced competitiveness and multiple mating of females	[47]
Use of sterile insects, conditional lethals, hybrid sterility etc.	[48]
Immigration of fertilized females and density dependence	[49]
***Genetic basis of sterility***	
Genetic and non-genetic sterility and early review of the screwworm programme	[50]
Genetic mechanisms involved in radiation sterilization	[51]
Genetics of radiation sterilization and review of genetic sexing strains	[32]
***Field programmes***	
Operational genetic control programmes for vectors	[52]
Field programme successes and failures	[53]
Fruit fly eradication in Japan	[54]
***SIT and transgenesis***	
Inducing molecular sterility using genetic transformation	[55]
Integrating SIT with a transgenic release and review of mosquito SIT programmes	[8]
Modelling the use of molecular sterility	[56]
